# Internal Tissue Expansion Induces Outward Migration of ADSCs From the Subcutaneous Fat Flap to Promote Skin Regeneration of the Expanded Area

**DOI:** 10.1155/sci/8680042

**Published:** 2025-12-23

**Authors:** Haojing Tang, Zhixin Xue, Ye Li, Ziqing Dong, Yunjun Liao

**Affiliations:** ^1^ Department of Plastic and Cosmetic Surgery, Nanfang Hospital, Southern Medical University, Guangzhou, Guangdong, China, fimmu.com

**Keywords:** ADSC, breast reconstruction, stem cell migration, tissue expansion, tissue regeneration

## Abstract

Repair of large soft tissue defects by tissue expansion often faces difficulties in skin expansion. Particularly in those who have lost a significant amount of skin and subcutaneous tissue due to total mastectomy, tissue expansion may result in skin breakdown and exposure of the expander. Subcutaneous fat construction by autologous fat grafting before expansion seems to assist skin expansion. We hypothesize that it may be related to the adipose‐derived stem cells (ADSCs) in subcutaneous fat. In this study, we confirmed this phenomenon through animal experiments and provided a preliminary investigation of the possible mechanisms involved. Four groups were designed for the experiment, experimental group (EG) for autologous fat flap transfer and tissue expansion. Fat grafting control group (FGCG), where only autologous fat flap transfer was performed without tissue expansion. Tissue expansion control group (TECG) did not perform autologous fat flap transfer but only skin tissue expansion on the back. Blank control group (BCG) has not received any surgery. In each group of 20 rats, skin, and fat flaps of the dilated area were sampled at four time points (7, 14, 21, and 28 days, *n* = 5), and the immediate skin retraction rate of the expanded area in the EG and TECG groups was measured to evaluate the expansion effect. GFP^+^ADSCs were observed to demonstrate whether they were facilitated by tissue expansion and migrated from autologous fat flaps to skin. Our study supported that fat layer construction prior to skin expansion helps to promote skin growth. The promotion may be related to the migration of ADSCs from adipose to dermis by compression, and ADSCs migrating to dermis further promote skin stretching through direct differentiation or paracrine cytokines to promote cell proliferation and collagen synthesis.

## 1. Background

Total mastectomy for breast cancer often results in significant loss of skin and subcutaneous tissue, posing challenges for reconstruction. Implant‐based breast reconstruction combined with fat grafting has emerged as a promising approach due to its minimal scarring and favorable cosmetic outcomes [1]. While immediate implant‐based reconstruction is typically suitable for patients undergoing breast‐conserving surgery with adequate residual skin [2], it is problematic for patients undergoing total mastectomy due to insufficient skin integrity, increasing the risk of complications such as skin breakdown and implant exposure [2–4]. Various strategies have been proposed to reinforce soft tissue support and minimize these complications. Initial methods, such as subpectoral implant placement, revealed practical limitations, including pain, compromised cosmetic outcomes, and dependence on muscle integrity [5]. Alternatives like partial muscle coverage combined with tissue‐engineered matrices have offered incremental improvements but have not entirely resolved these issues [6, 7]. Recently, implant placement above the pectoralis major muscle using porcine acellular dermal matrix (PADM), relying solely on subcutaneous tissue support, has been proposed as an effective alternative [8, 9].

In our clinical practice, we explored a novel approach combining intradermal expansion with fat grafting. We hypothesized that preinjecting autologous fat beneath the skin could reinforce soft tissue thickness and serve as a protective barrier during expansion, facilitating more effective skin growth and reducing expansion duration. Supporting evidence from previous research suggests that autologous fat layers not only physically protect the expanding skin but may also accelerate skin regeneration processes [10–14].

Adipose tissue is rich in adipose‐derived stem cells (ADSCs), known for their regenerative and wound‐healing properties [15]. We further investigated this mechanism in a rat intradermal expansion model, demonstrating that ADSCs within the graft migrate into the dermis under mechanical stress, enhancing skin regeneration. Additionally, gene expression analyses from public datasets allowed us to identify possible molecular mechanisms underlying this ADSC‐mediated skin regeneration response.

## 2. Methods

### 2.1. Animals

All experimental animals in this research project were purchased from the Laboratory Animal Center of Southern Medical University. Animal experiments were approved by the Animal Ethics Committee of Nanfang Hospital and conducted in compliance with the guidelines of the National Health and Medical Research Council (China). Experimental rats were housed in groups of four per cage under specific‐pathogen‐free (SPF) conditions with a 12‐h light–dark cycle. Animals were provided a standard laboratory diet and water ad libitum, without exposure to adverse environmental factors. Rats underwent a 1‐week acclimation period prior to initiating experiments.

### 2.2. Surgery

Anesthesia was induced in Sprague‐Dawley (SD) rats with 2–3 L/min of isoflurane, maintained at 500–700 mL/min via mask inhalation. After adequate preparation and disinfection of the surgical site, a 2–3 cm incision was made above the right inguinal region. The inguinal fat flap was meticulously dissected using blunt techniques while preserving the main vasculature. After suturing the incision, the outline of the transferred fat flap was marked externally. Rats were returned to their cages upon full recovery from anesthesia. For rats undergoing tissue expansion, a silicone tissue expander (Guangzhou Wanhe Plastic Material Co., Ltd.; 10 mL cylindrical expander, 33 × 22 mm, sterilized by cobalt‐60 irradiation) was placed subcutaneously between the fat flap and underlying muscle. Postplacement, the incision was sutured, the flap area marked, and the expander externally secured to prevent displacement (Figure 1A).

Figure 1(A, B) Schematic diagram of animal modeling and grouping. (C–E) The changes of epidermal and dermal histology and thickness in the EG group, FGCG group, TECG group, and BCG group were analyzed by HE staining after 7, 14, 21, and 28 days. Epidermis (μm, mean ± SD): 7 days: EG 34.55 ± 6.58, FGCG 41.99 ± 6.84, TECG 36.26 ± 8.55, BCG 35.11 ± 9.79; 14 days: EG 28.45 ± 5.48, FGCG 41.82 ± 9.12, TECG 29.40 ± 8.16, BCG 36.96 ± 7.54; 21 days: EG 31.77 ± 12.76, FGCG 35.25 ± 7.97, TECG 30.87 ± 8.64, BCG 34.64 ± 9.76; 28 days: EG 30.04 ± 6.03, FGCG 44.10 ± 7.99, TECG 33.34 ± 6.58, BCG 36.17 ± 8.44; Dermis (μm, mean ± SD): 7 days: EG 1931.45 ± 111.34, FGCG 1217.66 ± 101.92, TECG 1601.52 ± 96.26, BCG 1268.14 ± 81.57; 14 days: EG 1805.88 ± 107.46, FGCG 1554.93 ± 132.90, TECG 1670.97 ± 119.05, BCG 1457.18 ± 129.40; 21 days: EG 2048.89 ± 98.41, FGCG 1357.21 ± 52.30, TECG 1733.45 ± 27.68, BCG 1221.05 ± 104.35; 28 days: EG 1760.23 ± 166.21, FGCG 1386.37 ± 59.94, TECG 1607.98 ± 52.13, BCG 1235.40 ± 116.61. (F–J) Western blot analysis of the expression of dermis col III in the EG group, FGCG group, TECG group, and BCG group after 7, 14, 21, and 28 days. ns indicates no significant difference (*p* ≥ 0.05);  ^∗^ indicates *p* < 0.05,  ^∗∗^ indicates *p* < 0.01,  ^∗∗∗^ indicates *p* < 0.001, and  ^∗∗∗∗^ indicates *p* < 0.0001.

**Figure:**
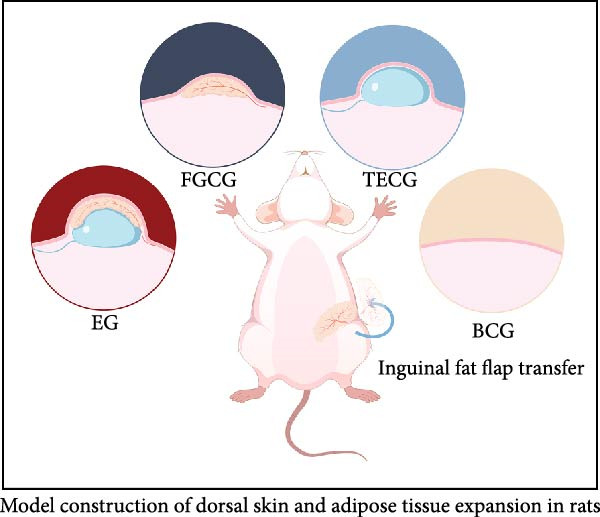
(A)

**Figure:**
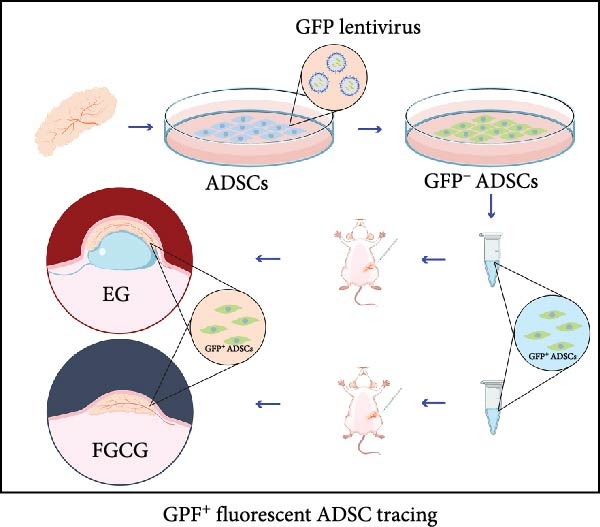
(B)

**Figure:**
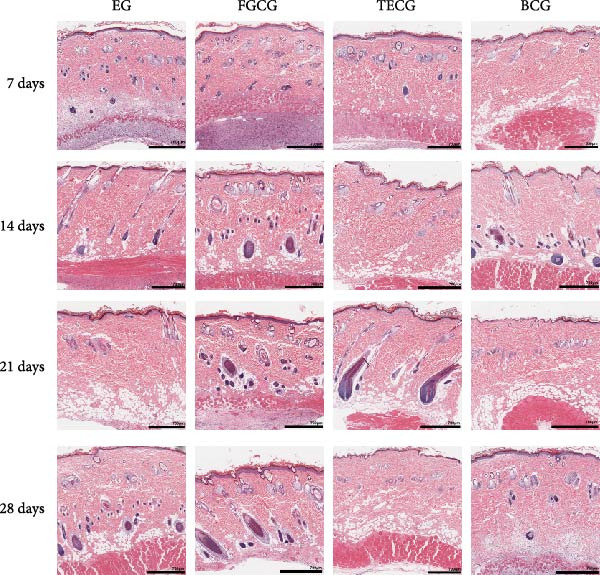
(C)

**Figure:**
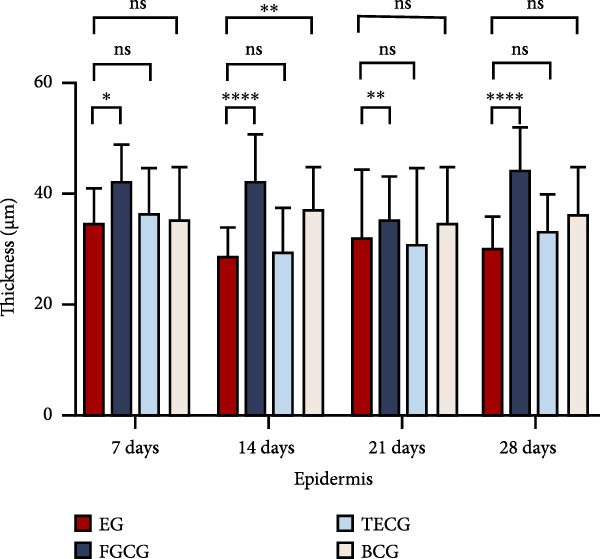
(D)

**Figure:**
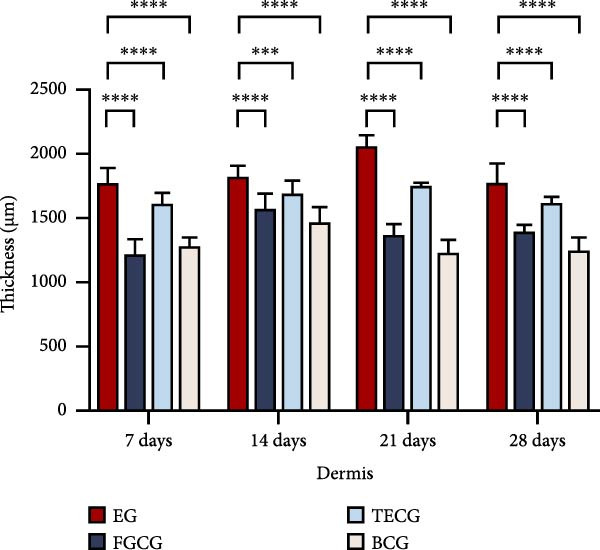
(E)

**Figure:**
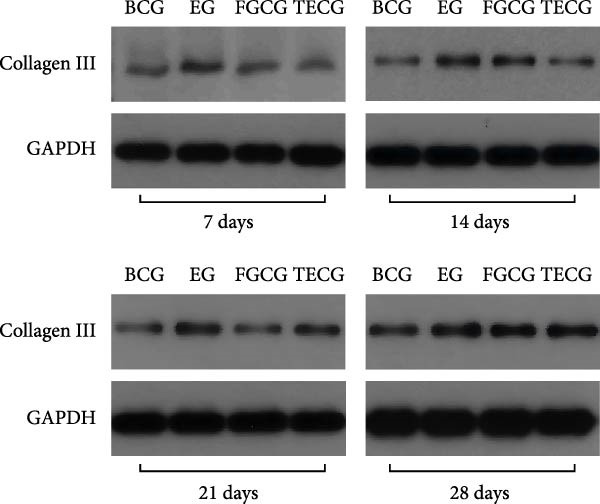
(F)

**Figure:**
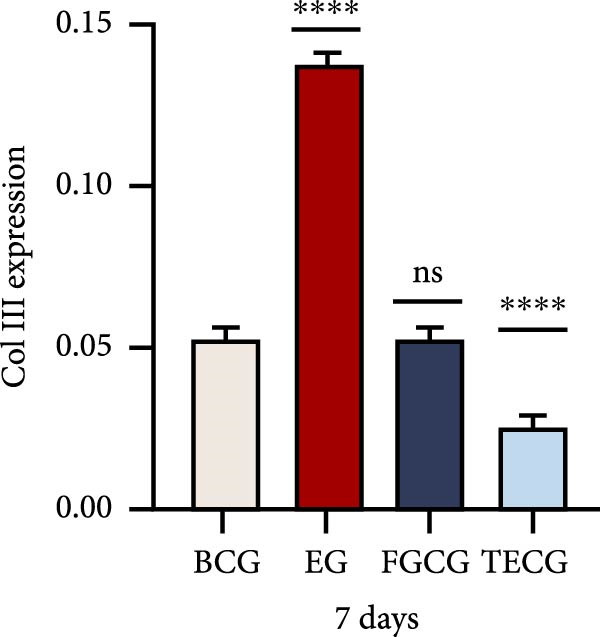
(G)

**Figure:**
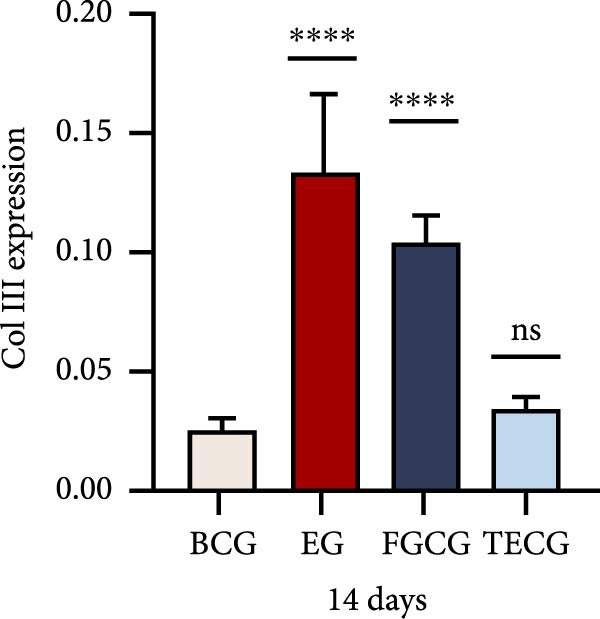
(H)

**Figure:**
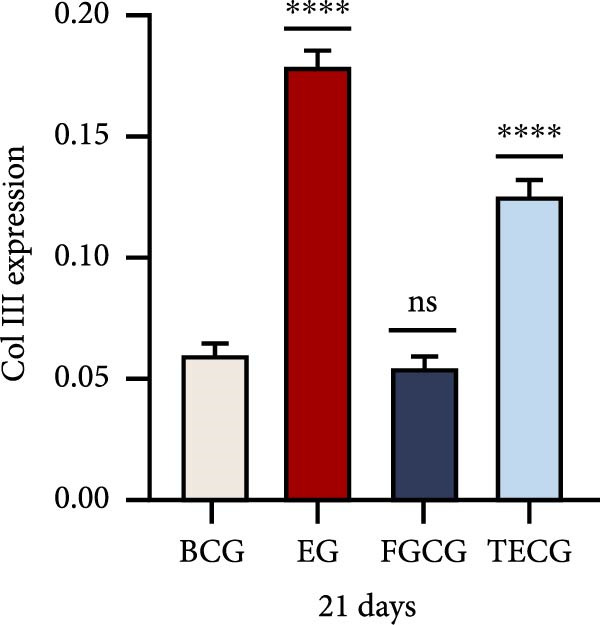
(I)

**Figure:**
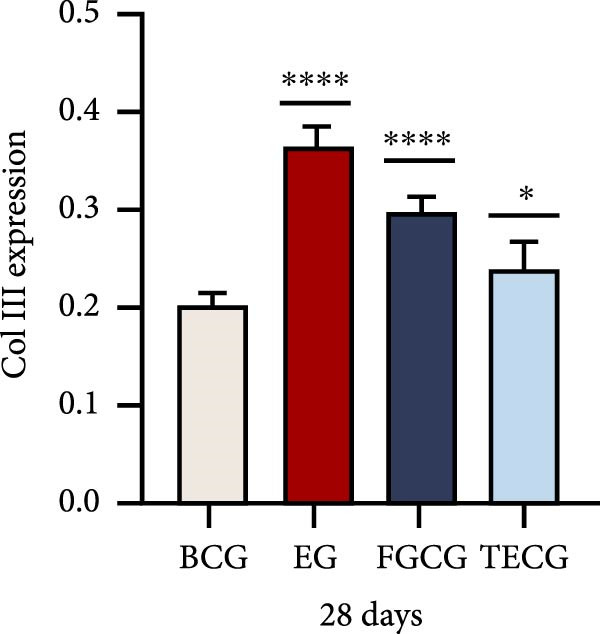
(J)

### 2.3. Experimental Group (EG) Design

Four EGs were established (Figure 1A): (1) EG underwent both autologous fat flap transplantation and tissue expansion. A 3 cm transverse incision was created mid‐dorsally, and the subcutaneous layer beneath the transplanted fat flap was bluntly dissected for tissue expander placement. The expander was inflated with 10 mL saline postsuturing. (2) Fat grafting control group (FGCG) underwent only fat flap transplantation without tissue expansion. (3) Tissue expansion control group (TECG) received only tissue expansion without fat flap transplantation. (4) Blank control group (BCG), consisting of age‐matched rats, did not undergo any surgical intervention. Each group comprised 20 rats, with samples collected from expanded skin and fat flaps at four time points (days 7, 14, 21, and 28; *n* = 5 per time point). The immediate skin retraction rate in EG and TECG groups was measured to assess tissue expansion efficacy. Tissue samples were either fixed in 4% paraformaldehyde for 48 h or stored at −80°C until further analysis.

### 2.4. Hematoxylin–Eosin (HE) Staining

Paraffin‐embedded tissue sections were deparaffinized and hydrated through sequential xylene and alcohol baths. Sections were stained with hematoxylin, rinsed under running water for 5 min, immersed in distilled water for 15 min, stained with eosin, dehydrated, and sealed. The slides were scanned using a Shenzhen Biostrength SQS40P slide scanning imaging system.

### 2.5. Immunohistochemical Staining

Antigen retrieval was performed using EDTA buffer (1:50 dilution). Endogenous peroxidase activity was blocked with hydrogen peroxide. Sections were incubated with anti‐proliferating cell nuclear antigen (PCNA) primary antibody (1:1000; Abcam, USA) for 12–24 h, followed by secondary incubation with sheep anti‐rabbit IgG (Santa Cruz Biotechnology). Diaminobenzidine (DAB; Thermo Scientific) was used for color development, and nuclei were counterstained with hematoxylin. Slides were dehydrated, cleared, and sealed with neutral gum.

### 2.6. Immunofluorescence Staining

Following EDTA‐mediated antigen retrieval (1:50 dilution) and hydrogen peroxide blocking, sections were incubated with primary antibodies anti‐α‐SMA (1:400), anti‐CD34 (1:200), and anti‐CD31 (1:200; all from Abcam, USA) for 12–24 h. Afterward, sections were treated with fluorescently labeled secondary antibodies and counterstained with DAPI (Thermo Scientific). Samples were mounted with ProLong Gold Antifade Mountant (Thermo Scientific).

### 2.7. Western Blotting

Protein samples were separated by gel electrophoresis and transferred onto membranes, which were then incubated overnight in blocking solution. Membranes were probed sequentially with primary antibodies against Collagen III (SAB) and HRP‐conjugated sheep anti‐mouse IgG secondary antibodies. Protein bands were visualized using enhanced chemiluminescence (ECL), and densitometric analysis was conducted using SensiAnsys software on a JS‐680A automatic gel imaging analyzer.

### 2.8. ADSC Culture and GFP Fluorescent Protein Transfection

ADSCs were isolated from the inguinal adipose tissue of two 6‐week‐old SD rats. Fat tissues were dissected, vascular components removed, minced, and enzymatically digested with collagenase I (Solarbio) at 37°C for 40 min. Digested tissues were centrifuged (180 *g*, 5 min), filtered, resuspended in erythrocyte lysis buffer (erythrocyte lysate:PBS ratio 4:1), and recentrifuged. The resultant cell pellet was cultured in adipose stem cell‐specific medium (containing 10% serum and 2% antibiotic–antimycotic solution). Cells were seeded into standard culture medium for subsequent expansion (Figure 2C).

Figure 2(A, B) Immunofluorescence staining was used to analyze the changes in the number of CD34^+^ cells in the dermis of the EG group, FGCG group, TECG group, and BCG group after 7, 14, 21, and 28 days. Quantification (%, mean ± SD): 7 days: EG 9.12 ± 2.24, FGCG 1.27 ± 1.01, TECG 1.26 ± 1.09, BCG 0.81 ± 0.49; 14 days: EG 17.08 ± 2.96, FGCG 1.74 ± 1.89, TECG 0.87 ± 0.58, BCG 0.79 ± 0.89; 21 days: EG 12.22 ± 4.00, FGCG 1.35 ± 0.76, TECG 1.14 ± 0.85, BCG 0.92 ± 1.04; 28 days: EG 7.17 ± 3.60, FGCG 2.21 ± 0.91, TECG 7.85 ± 3.03, BCG 1.64 ± 1.75. (C) GFP^+^ADSCs photographed by fluorescence inverted microscopy; (D) GFP^+^ADSCs were rarely seen in the dermis of FGCG group; (E (e)) Numerous GFP^+^ADSCs were seen in the dermis of EG group; (F) Numerous GFP^+^ADSCs were seen in the adipose of FGCG group; (G) Significant reduction of ADSCs in adipose of EG group; (H, I) Semi‐quantitative of GFP^+^ADSCs. ns indicates no significant difference (*p* ≥ 0.05);  ^∗^ indicates *p* < 0.05,  ^∗∗^ indicates *p* < 0.01,  ^∗∗∗^ indicates *p* < 0.001, and  ^∗∗∗∗^ indicates *p* < 0.0001.

**Figure:**
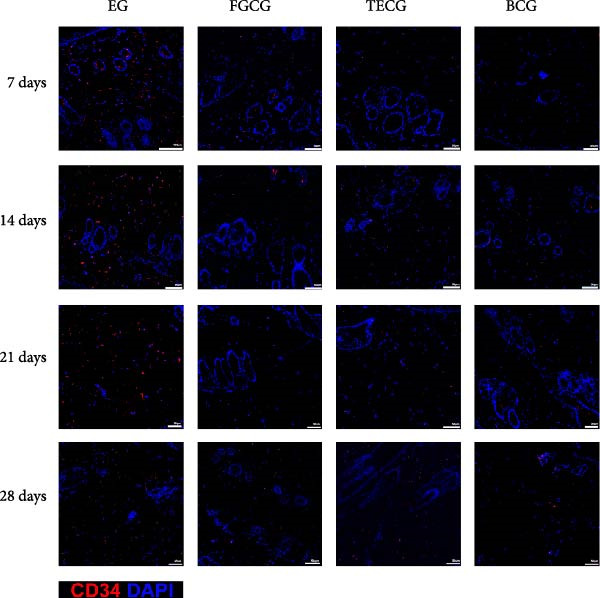
(A)

**Figure:**
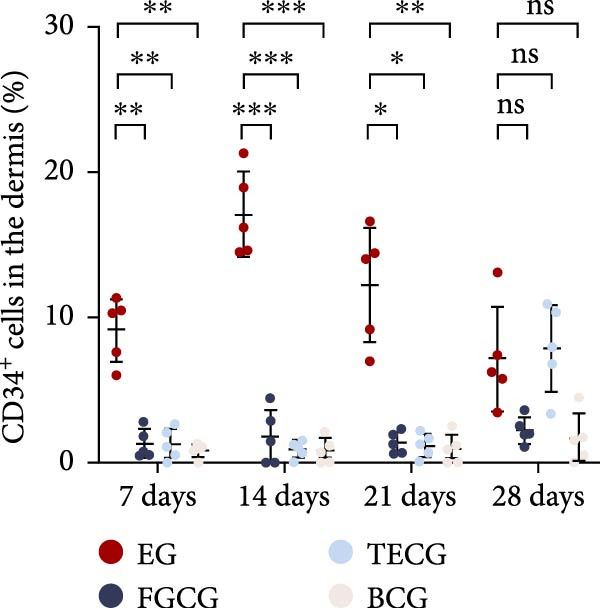
(B)

**Figure:**
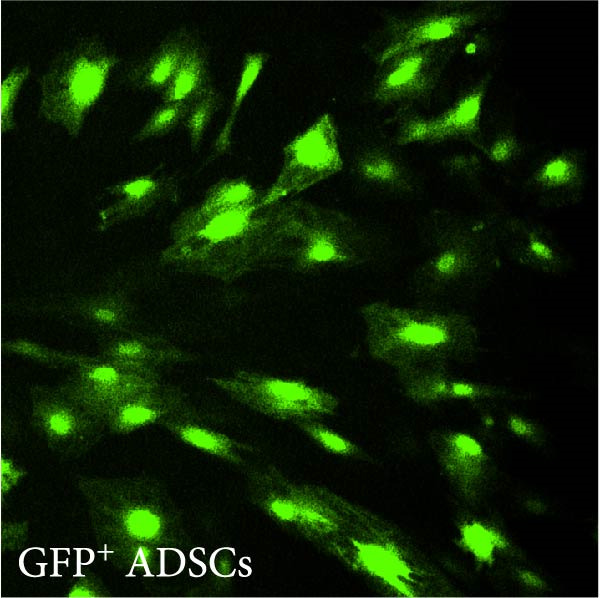
(C)

**Figure:**
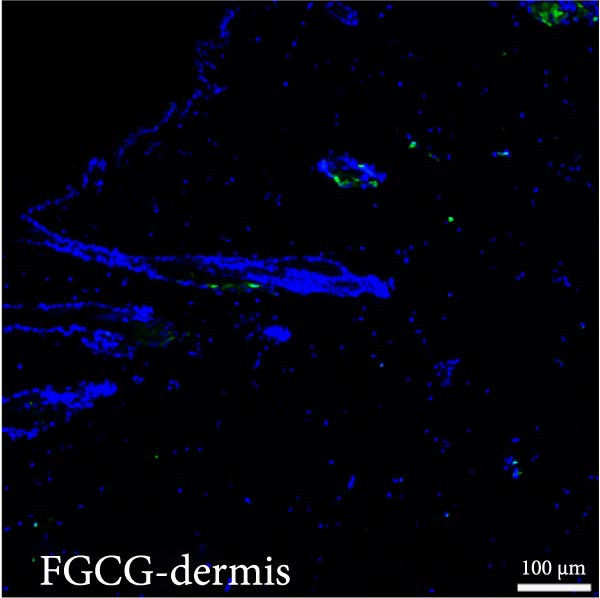
(D)

**Figure:**
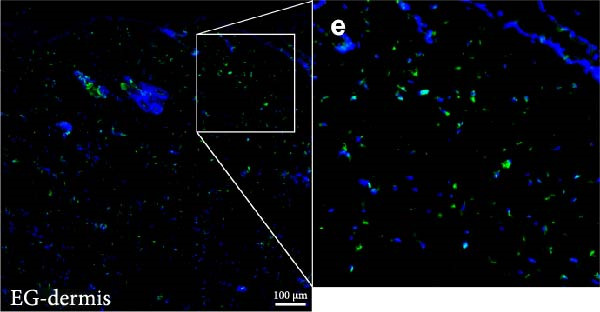
(E)

**Figure:**
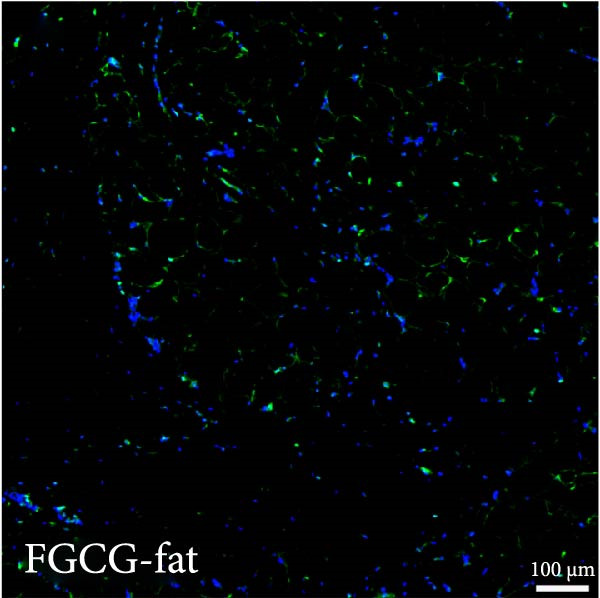
(F)

**Figure:**
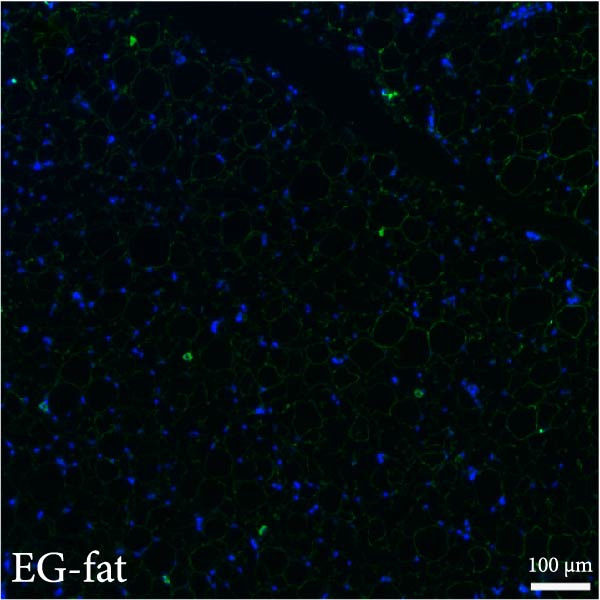
(G)

**Figure:**
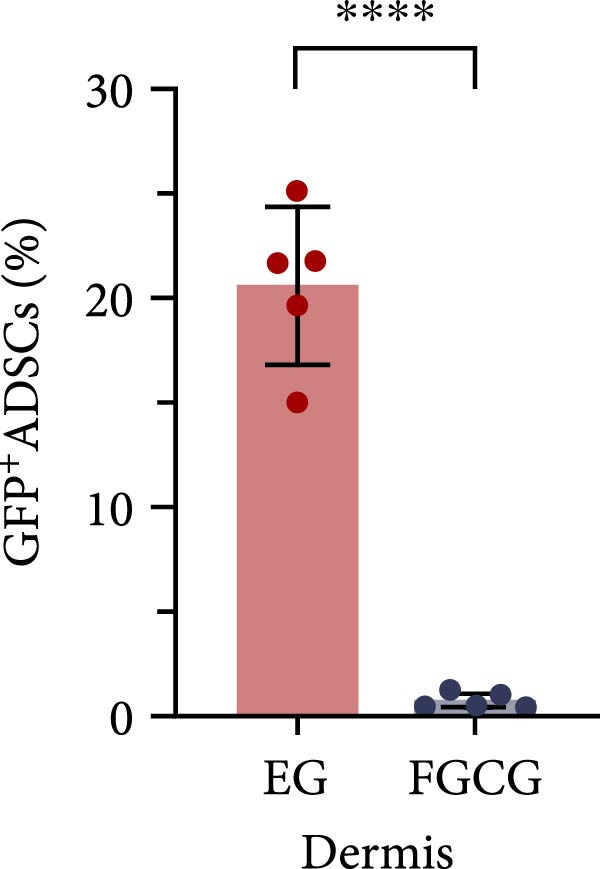
(H)

**Figure:**
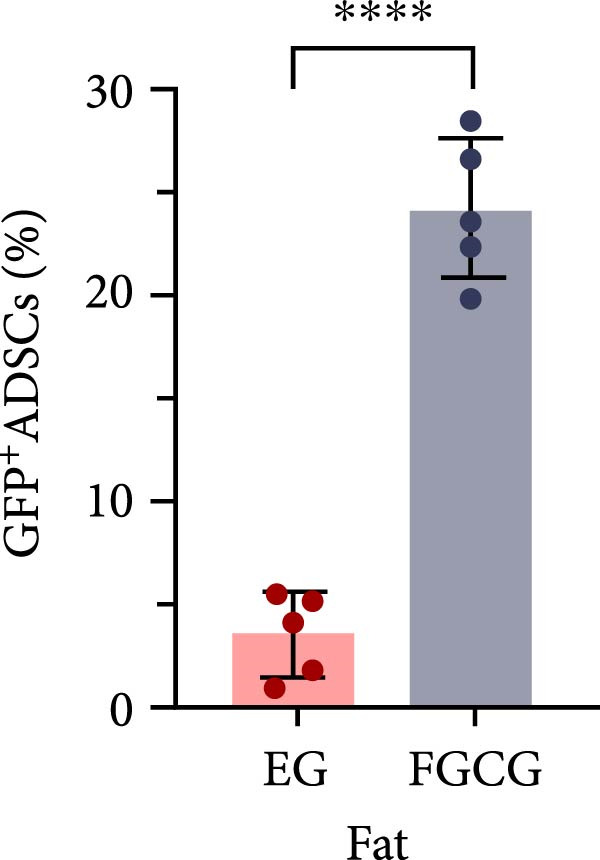
(I)

For transfection, ADSCs were plated in 6‐well plates (5 × 10^4^ cells/well). GFP lentivirus suspension (1 × 10^8^ TU/mL, 100 μL) mixed with culture medium containing Polybrene (5 μg/mL, total volume 1.5 mL) was added. Medium was refreshed after 12 h. GFP expression in ADSCs (>90%) was confirmed by fluorescence microscopy after 48 h. GFP‐positive ADSCs (1 × 10^5^ cells/rat) were reimplanted into rat inguinal adipose tissue for subsequent analysis (Figure 1B).

### 2.9. Statistical Analysis

All quantitative data are presented as mean ± standard deviation (SD). The experimental unit was the animal unless otherwise stated. For histology and immunostaining (HE thickness measures; α‐SMA+, CD34+, and CD31+ cell counts; PCNA‐positive area), ≥3 nonoverlapping microscopic fields per section were quantified and averaged to generate one value per animal for analysis. Western blot band intensities were normalized to the loading control and then to the mean of the BCG at the corresponding time point. The immediate skin retraction rate was calculated from the percentage change of the marked grid length after natural recoil; lower values indicate more effective expansion.

Normality was assessed with the Shapiro–Wilk test and homogeneity of variance with Levene’s test. For outcomes measured across groups and time points, a two‐way factorial ANOVA (factors: group [EG, FGCG, TECG, BCG] and time [days 7, 14, 21, 28]) was performed;

All statistical analyses were performed using GraphPad Prism (v9 or later), IBM SPSS Statistics (v26 or later), and R (v4.2+). SPSS was used to verify assumption checks (Shapiro–Wilk and Levene’s tests) and to replicate primary inferential analyses; results were consistent across platforms. Figures display individual data points with group means ± SD, and exact *p* values are reported where feasible.

### 2.10. Differentially Expressed Genes (DEGs) and Enrichment Analysis

The GSE189845 dataset was retrieved from the GEO database, with gene identifiers annotated via platform annotation data. Data preprocessing in RStudio yielded a standardized gene matrix devoid of duplicates and missing values. Samples were grouped into cyclic mechanical stretch (CMS) and non‐CMS based on CMS pretreatment. DEGs were identified and visualized using heatmaps. Annotated DEGs underwent Gene Ontology (GO) and Kyoto Encyclopedia of Genes and Genomes (KEGG) enrichment analyses using enrich GO and enrich KEGG functions, respectively, and results were visualized with bubble plots. DEGs and gene sets associated with cell migration, wound healing, and tissue regeneration were analyzed using STRING for protein–protein interaction (PPI) network construction.

## 3. Result

### 3.1. Significant Increases of Dermal Thickness Were Observed in the EG Group

Figure 1C demonstrates the HE staining of the whole skin layers of the four groups, EG, FGCG, TECG, and BCG, after 7, 14, 21, and 28 days. The measured mean thickness of the epidermis and dermis at each time point for each group is shown in Figure 1D,E. The epidermis of the EG and TECG groups, which underwent tissue expansion, had a thinner mean thickness of the epidermis compared to the FGCG and BCG groups, which did not undergo tissue expansion (e.g., at 14 days: EG 28.45 ± 5.48 and TECG 29.40 ± 8.16 vs. FGCG 41.82 ± 9.12 and BCG 36.96 ± 7.54; at 28 days: EG 30.04 ± 6.03 and TECG 33.34 ± 6.58 vs. FGCG 44.10 ± 7.99 and BCG 36.17 ± 8.44). However, the pattern of epidermal thickness over time was not significant, but the dermis of the EG and TECG groups was significantly thicker compared with the FGCG and BCG groups without tissue expansion (e.g., at 21 days: EG 2048.89 ± 98.41 and TECG 1733.45 ± 27.68 vs. FGCG 1357.21 ± 52.30 and BCG 1221.05 ± 104.35). Apart from this, the dermal thickness was significantly higher in the EG group than in the TECG group at any time point (*p*  < 0.05).

### 3.2. Western Blotting Detected a Significant Increase of Dermal Collagen III Expression in the EG Group

Figure 1F–J shows the expression level of type III collagen in the dermis of each group at each time point. Using BCG, overall, there was a significant increase in the expression level of type III collagen in the EG group (e.g., day 7: EG 0.143 ± 0.016 vs. BCG 0.054 ± 0.004; day 14: EG 0.125 ± 0.008 vs. BCG 0.026 ± 0.004). The elevated trend in the EG group could be seen from the seventh day (e.g., day 7: EG 0.143 ± 0.016 vs. BCG 0.054 ± 0.004). And a significant elevation of type III collagen expression was also observed in the TECG group on the following 21 days (e.g., day 21: TECG 0.127 ± 0.011 vs. BCG 0.060 ± 0.007). The FGCG group, on the other hand, observed a significant elevation of type III collagen expression on the 21st and 14th days (*p*  < 0.05) (e.g., day 14: FGCG 0.104 ± 0.007 vs. BCG 0.026 ± 0.004).

### 3.3. Immunofluorescence Showed a Significant Increase in the Number of α‐SMA^+^ Cells in the EG Group

The fluorescence costaining of α‐SMA and DAPI in skin staining maps of EG, FGCG, TECG, and BCG groups after 7, 14, 21, and 28 days, respectively, is shown in Figure 3. Semi‐quantitative results showed that the number of α‐SMA + cells in the EG group was significantly higher than that in the other groups (*p*  < 0.05) (e.g., at 21 days: EG 22.25 ± 4.62 vs. FGCG 1.27 ± 0.76, TECG 6.21 ± 2.84, BCG 0.54 ± 0.32); observing the trend of the cell number over time, the number of α‐SMA^+^ cells in the EG group began to rise after 7 days and reached the peak after 21 days before beginning to decline (e.g., day 7: 6.84 ± 2.57; day 21: 22.25 ± 4.62; day 28: 9.29 ± 3.43). The TECG group also showed a trend of rising over time, but at a later rate than the EG group and with a smaller number than the EG group (e.g., day 21: TECG 6.21 ± 2.84 vs. EG 22.25 ± 4.62; day 28: TECG 8.30 ± 3.33). α‐SMA^+^ cells were also observed in the FECG group, but the overall level was lower than that in the EG and TECG groups (e.g., day 14: FGCG 1.31 ± 0.76 vs. BCG 1.02 ± 0.37; day 21: FGCG 1.27 ± 0.76 vs. BCG 0.54 ± 0.32).

Figure 3(A) Immunofluorescence staining was shown to analyze the changes in the number of α‐SMA + cells in the dermis of the EG group, FGCG group, TECG group, and BCG group after 7, 14, 21, and 28 days. (B) Quantification (%, mean ± SD): 7 days: EG 6.84 ± 2.57, FGCG 0.69 ± 0.47, TECG 1.76 ± 0.52, BCG 0.67 ± 0.49; 14 days: EG 9.37 ± 3.21, FGCG 1.31 ± 0.76, TECG 2.55 ± 1.71, BCG 1.02 ± 0.37; 21 days: EG 22.25 ± 4.62, FGCG 1.27 ± 0.76, TECG 6.21 ± 2.84, BCG 0.54 ± 0.32; 28 days: EG 9.29 ± 3.43, FGCG 0.62 ± 0.55, TECG 8.30 ± 3.33, BCG 0.85 ± 0.41 (*p*  < 0.05). ns indicates no significant difference (*p* ≥ 0.05);  ^∗^ indicates *p* < 0.05,  ^∗∗^ indicates *p* < 0.01.

**Figure:**
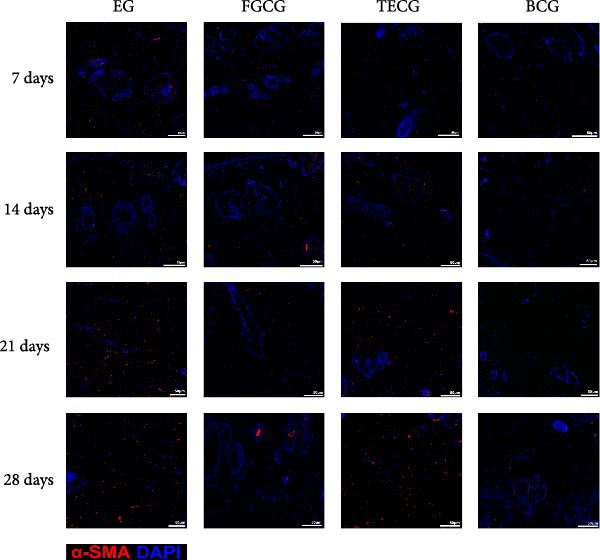
(A)

**Figure:**
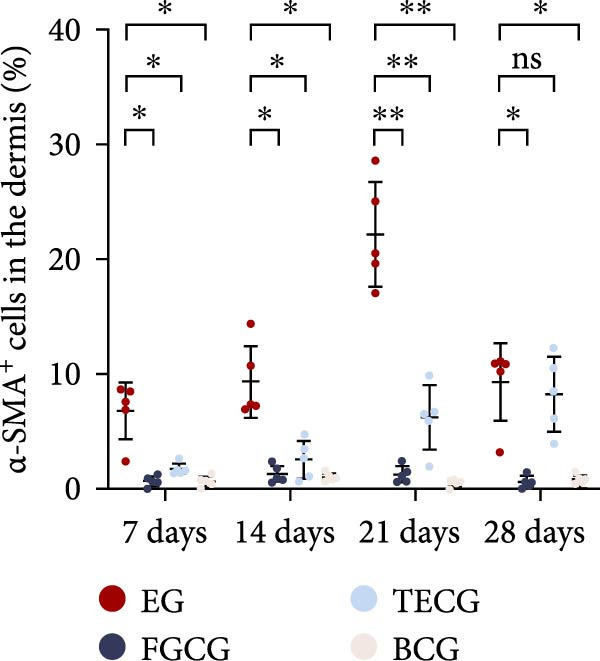
(B)

### 3.4. PCNA Was Significantly Expressed in the Dermis of EG Group

Immunohistochemical staining of PCNA taken from EG, FGCG, TECG, and BCG groups after 14 days is shown in Figure 4A–D. The semi‐quantitative results of Figure 4E showed that the positive area of PCNA in the dermis of EG group was significantly higher than that of the other groups (*p*  < 0.05) (e.g., EG 16.50 ± 4.52 vs. FGCG 1.76 ± 0.50, TECG 8.53 ± 3.63, and BCG 2.29 ± 1.71), which suggests that dermal cell proliferation was more vigorous in the EG group. The positive area of TECG was inferior to that of the EG group (*p*  < 0.05) (e.g., TECG 8.53 ± 3.63 vs. BCG 2.29 ± 1.71), but still had a significant increase compared with the BCG group (*p*  < 0.05). No significant difference was seen in FECG compared with BCG (*p*  > 0.05) (e.g., FGCG 1.76 ± 0.50 vs. BCG 2.29 ± 1.71).

Figure 4(A–E) Immunohistochemical staining was used to analyze the positive area of PNCA in the dermis of the EG group (16.50% ± 4.52%), FGCG group (1.76% ± 0.50%), TECG group (8.53% ± 3.63%), and BCG group (2.29 ± 1.71) after 14 days; (F–H) Skin retraction rate measurement compared skin growth in the dilated area of the EG and TECG groups after 7, 14, 21, and 28 days. Immediate skin retraction rate (%, mean ± SD): 7 days: EG 56.08 ± 3.65, TECG 59.76 ± 5.77; 14 days: EG 31.18 ± 5.61, TECG 55.62 ± 5.05; 21 days: EG 26.09 ± 2.70, TECG 47.57 ± 3.70; 28 days: EG 23.25 ± 4.61, TECG 33.25 ± 4.89 (lower retraction rate indicates better expansion; EG < TECG at days 14 and 21, *p*  < 0.05). ns indicates no significant difference (*p* ≥ 0.05);  ^∗^ indicates *p* < 0.05,  ^∗∗^ indicates *p* < 0.01.

**Figure:**
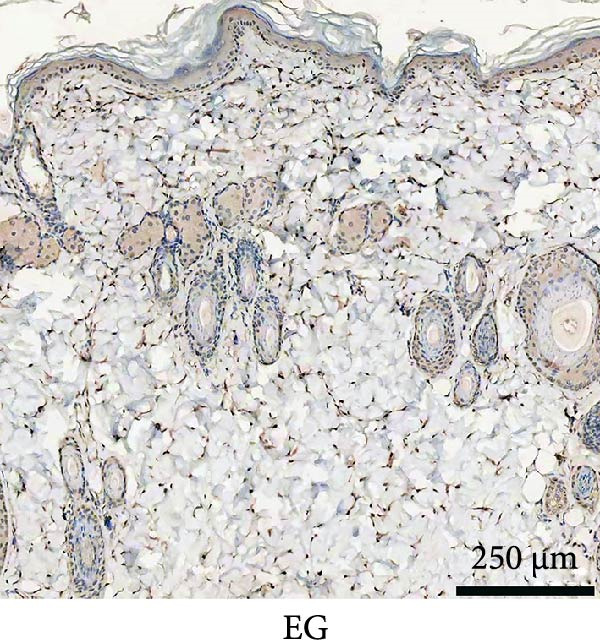
(A)

**Figure:**
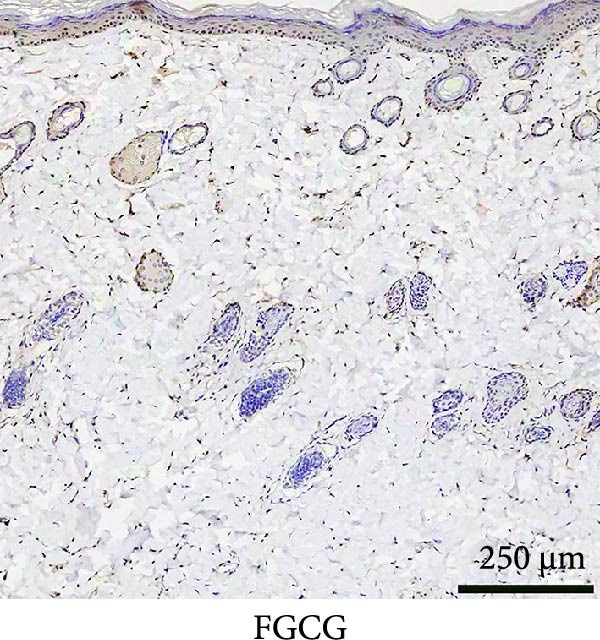
(B)

**Figure:**
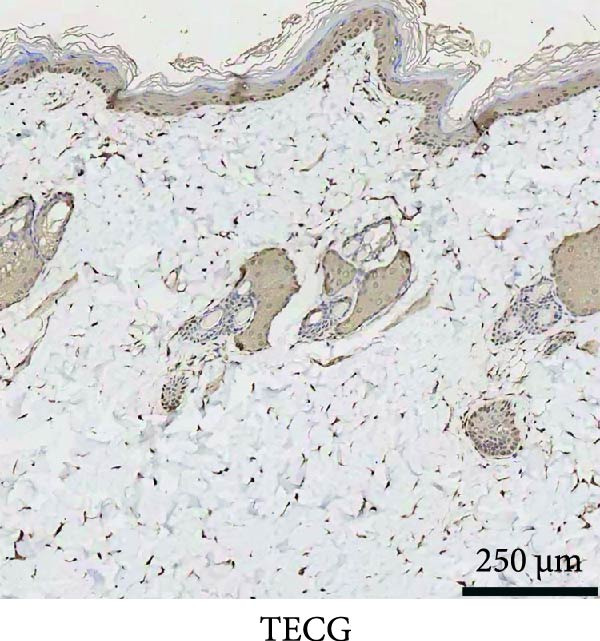
(C)

**Figure:**
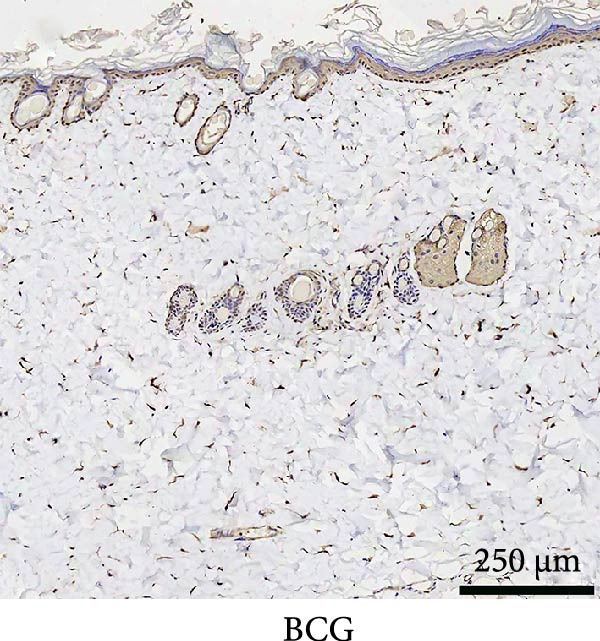
(D)

**Figure:**
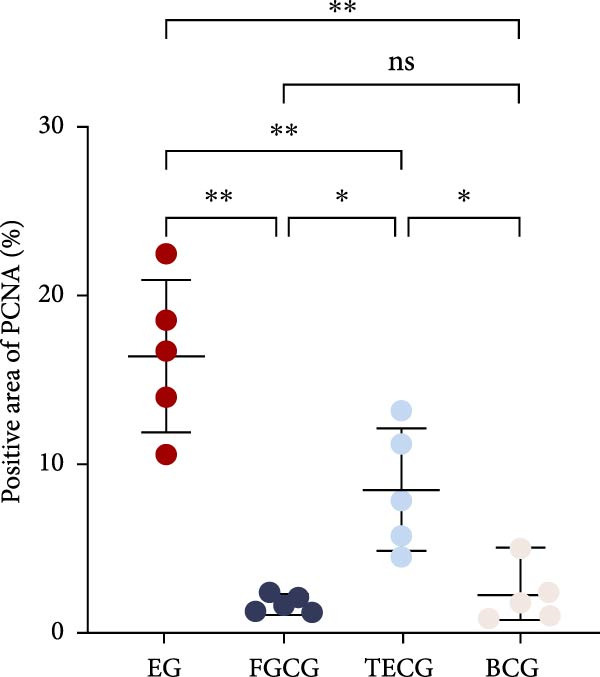
(E)

**Figure:**
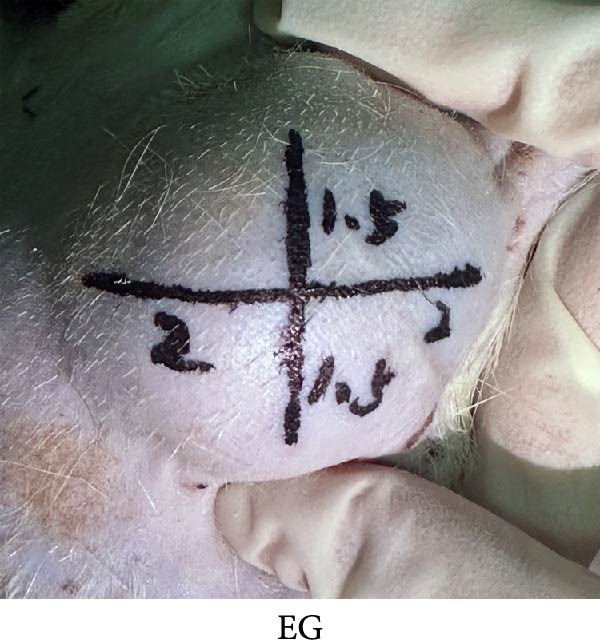
(F)

**Figure:**
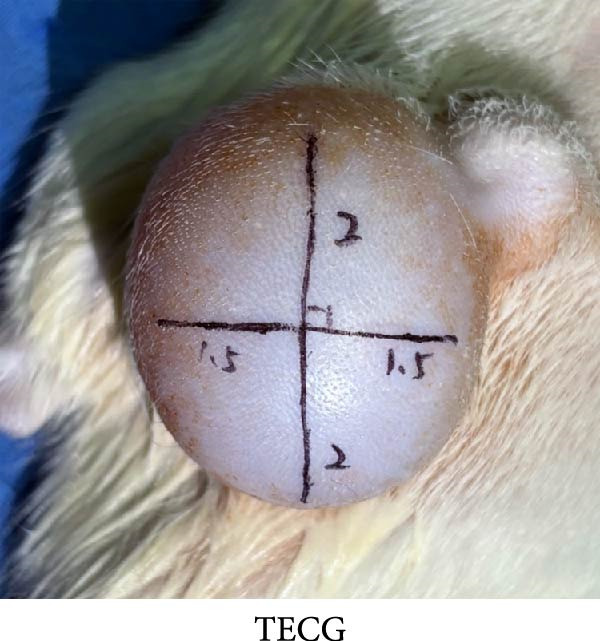
(G)

**Figure:**
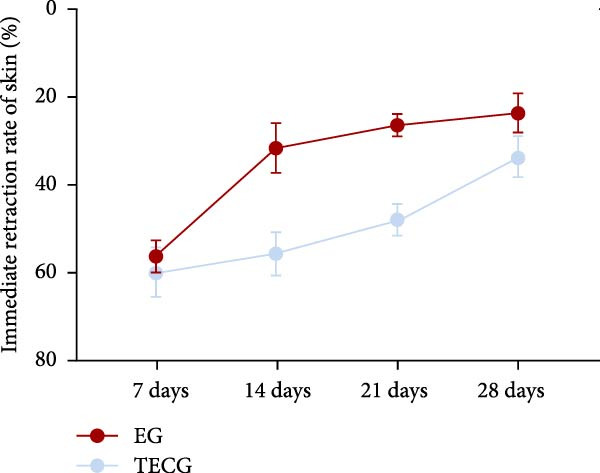
(H)

### 3.5. Measurement of the Immediate Skin Retraction Rate Showed That the Early Skin Expansion Efficiency Was Higher in the EG Group Than in the TECG Group

Cross marks (3 × 4 cm) were done on the skin surface of the expanded area in the EG group and the TECG group (Figure 4F,G). The length of the marking line was measured again in the natural retraction state and calculated to calculate the immediate skin retraction rate. Lower retraction rate suggested a better skin expansion effect. Figure 4H results showed that the immediate skin retraction rate of the EG group was significantly smaller than that of the TECG group from the 14th to the 21st day of expansion (*p*  < 0.05) (e.g., at 14 days: EG 31.18 ± 5.61 vs. TECG 55.62 ± 5.05; at 21 days: EG 26.09 ± 2.70 vs. TECG 47.57 ± 3.70). The trend showed that the rate of increase in expansion efficiency was higher in the EG group than in the TECG group, suggesting that the presence of the subcutaneous fat flap promoted early tissue expansion efficiency.

### 3.6. Immunofluorescence Showed an Increase in the Number of CD34^+^ Cells in the Dermis of the EG Group

Figure 2A demonstrates the fluorescence costaining of CD34 and DAPI in the skin tissues of the four groups of EG, FGCG, TECG, and BCG on days 7, 14, 21, and 28. Semi‐quantitative results (Figure 2B) showed the proportion of CD34+ cells at each time point in each group. The results suggested that the number of CD34+ cells in the EG group was significantly higher than that in the other groups (*p*  < 0.05) (e.g., peak at day 14: EG 17.08 ± 2.96 vs. FGCG 1.74 ± 1.89, TECG 0.87 ± 0.58, BCG 0.79 ± 0.89; early at day 7: EG 9.12 ± 2.24 vs. BCG 0.81 ± 0.49); observing the trend of the cell number over time, the number of CD34^+^ cells in the EG group increased early over time and reached the peak on day 14. A significant increase in CD34+ cells in the TECG group compared to the BCG group was not observed (*p*_7_  > 0.05; *p*_14_  > 0.05; *p*_21_  > 0.05) until day 28 (*p*  < 0.05) (e.g., day 28: TECG 7.85 ± 3.03 vs. BCG 1.64 ± 1.75). CD34^+^ cells were also observed in the FECG group, but no significant difference was seen compared to the BCG group (*p*  > 0.05) (e.g., day 14: FGCG 1.74 ± 1.89 vs. BCG 0.79 ± 0.89).

### 3.7. Tissue Expansion Promotes Migration of GFP^+^ ADSCs From Adipose Flaps to the Dermis

Figure 2C shows GFP^+^ ADSCs cultured in vitro, taken under a fluorescence inverted microscope. 0.5 mL of cell suspension containing 5 × 10^5^ GFP^+^ ADSCs was injected into autologous fat flaps of SD rats in the EG and FGCG groups. The distribution of ADSCs within the fat flaps and the expanded skin flaps of the two groups of rats was observed at the 14th day, respectively. Semi‐quantitative results (Figure 2D–I) showed that a large number of GPF^+^ cells were observed in the dermis of the EG group (Figure 2E,e), which was significantly higher than that of the FGCG group (Figure 2D). There was a significant decrease in the GPF^+^ cells of the subcutaneous fat flap of the EG group (Figure 2G) as compared with that of the FGCG group (Figure 2F) (*p*  < 0.05). These findings suggest that in the EG group, a large proportion of the GFP^+^ ADSCs originally present in the subcutaneous fat flap migrated into the dermis of the expanded area. In contrast, in the FGCG group, little migration of GFP^+^ADSCs from the fat flap occurred.

### 3.8. Tissue Expansion Contributes to the Increase of Skin Blood Vessels of Expanded Skin

The distribution of blood vessels labeled by CD31 under immunofluorescence staining is shown as Figure 5A,B. The EG group on the 14th day showed a denser distribution of blood vessels in the dermis compared with the FGCG group (Figure 5D). A large number of GFP + cells could be observed in the EG group on day 7, distributed around the superficial dermal blood vessels, and GFP fluorescence signals were also seen in some of the blood vessels (Figure 5C). This suggested that the ADSCs of the fat flap might have migrated through the local vascular network of adipose and dermis (Figure 5E shows the macroscopic observation of newly formed local vascular communication between expanded skin and transferred fat flap). It suggests that expansion promotes the formation of a denser vascular network between adipose and dermis, providing more pathways for stem cell delivery and migration.

Figure 5(A, B, D) Immunofluorescence staining analysis showed the number of CD31‐labeled blood vessels in the dermis of the EG group and FGCG group on 14th day; (C) Immunofluorescence staining showed that a large number of GPF^+^ADSCs were distributed in the superficial dermal and perivascular vessels of the EG group after 7 days; (E) Macroscopic view of the newly formed local vascular communication between the expanded skin and the transferred fat flap; (F) Immunofluorescence costaining showed that some GFP and α‐SMA signals overlapped.  ^∗∗∗∗^ indicates *p* < 0.0001.

**Figure:**
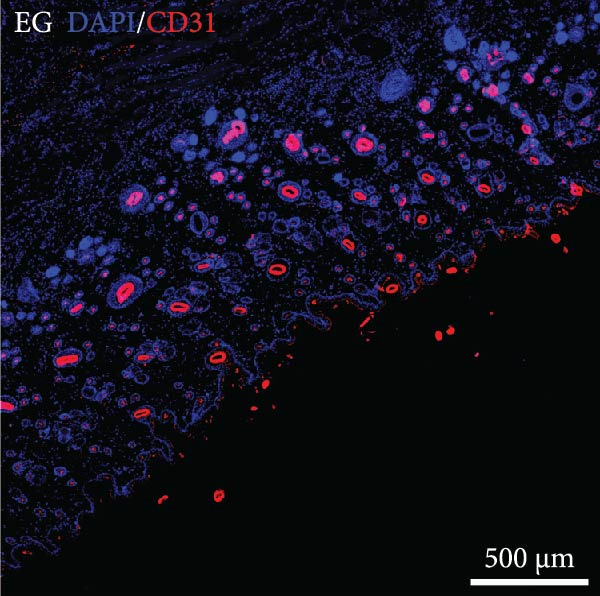
(A)

**Figure:**
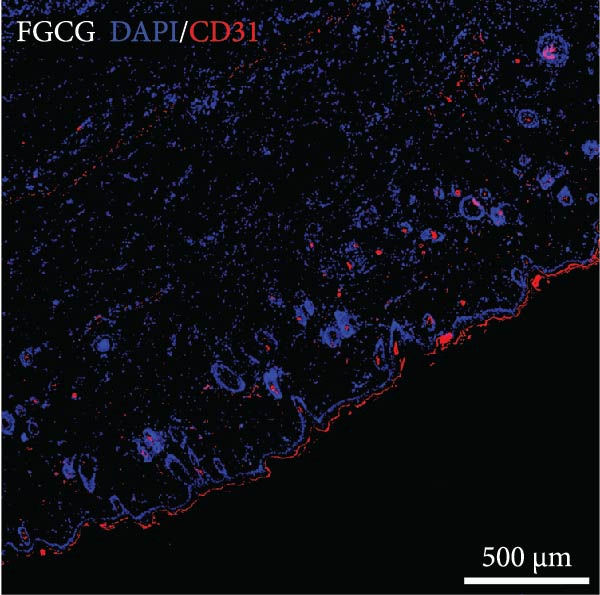
(B)

**Figure:**
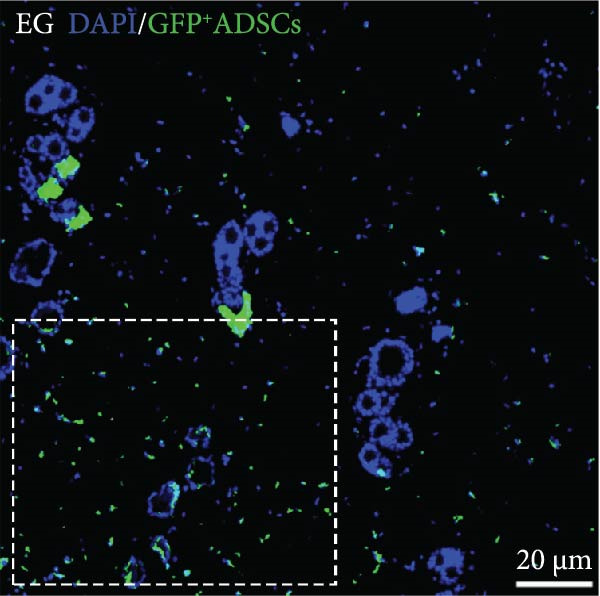
(C)

**Figure:**
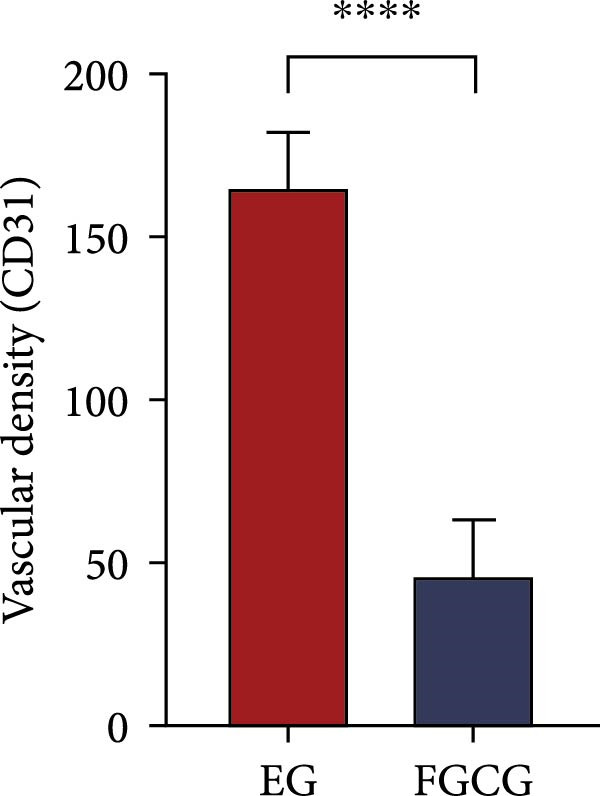
(D)

**Figure:**
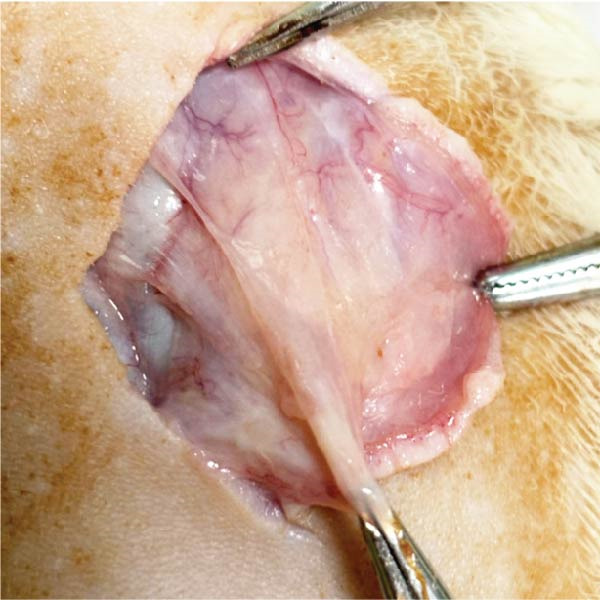
(E)

**Figure:**
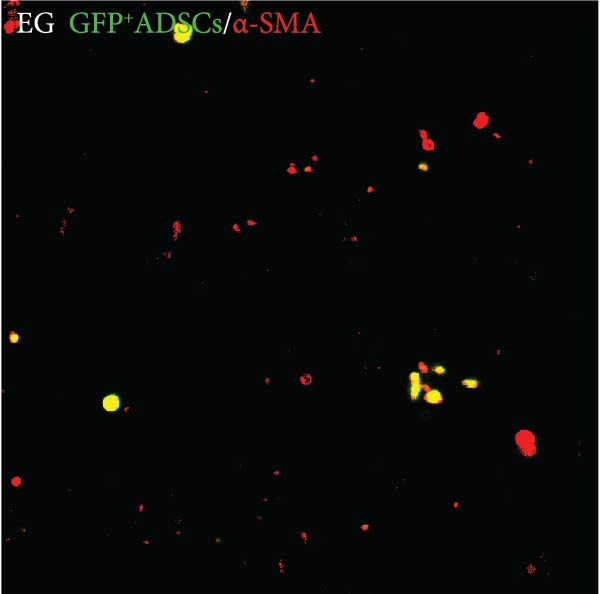
(F)

### 3.9. Some of the Implanted GFP^+^ Cells Also Express α‐SMA at the 14th Day

In the previous section, we found that the dermis of the EG group had a significant increase in α‐SMA + cells. To further explore the relationship between migrating stem cells and α‐SMA + cells, we costained α‐SMA with GFP fluorescence signals in the dermis of the EG group taken on day 14. Overlapping of GFP and α‐SMA signals was observed in some of the fields of view (Figure 5F). This suggests that some of the ADSCs that migrated from the adipose flap into the dermis differentiated directly into α‐SMA^+^ cells.

### 3.10. Transcriptomics Revealed Genes That Were Differentially Expressed in Mesenchymal Stem Cells (MSCs) After Mechanical Force Treatment In Vitro

By analyzing GSE189845 transcriptomics data, 2625 DEGs were identified (Figure 6A,B). Among them, 1282 genes were up‐regulated, in which, the top 10 genes ranked by fold change were GASK1A, NEFM, MLANA, ADAMTS16, AKR1C15, GBP6, AKR1C14, CADM3, ANKRD34C, and PTCHD1. 1343 genes were downregulated, and the top 10 genes ranked by fold change were *IL11*, *NPY*, *IL23R*, *LOC100909605*, *SERPINB3A*, *RGD1565462*, *LOC100912228*, *IL10*, *ZMIZ2*, and *GPR83*. GO enrichment analysis was performed on these DEGs to explore their functions (Figure 6C). These 2625 DEGs were mainly enriched in biological processes (BPs) in positive regulation of cytokine production, wound healing, leukocyte migration, response to oxygen levels, and extracellular matrix organization. In cellular components (CCs), they were mainly enriched in collagen‐containing extracellular matrix, cell‐base junctions, vacuolar membranes, focal adhesion, lysosomal membranes. In molecular function (MF), they were mainly enriched in receptor‐ligand activity, signaling receptor activator activity, sulfur compound binding, glycosaminoglycan binding, and extracellular matrix structure constituent.

Figure 6Heatmap (A) and volcano plot (B) demonstrate the differentially expressed genes (DEGs) in MSCs from CMS group and non‐CMS; (C) GO enrichment analysis of DEGs between CMS group and non‐CMS in MSCs; (D, E) KEGG pathway enrichment analysis and protein interaction analysis of DEGs enriched for cell migration; (F, G) KEGG pathway enrichment analysis and protein interaction analysis of DEGs enriched in wound healing.

**Figure:**
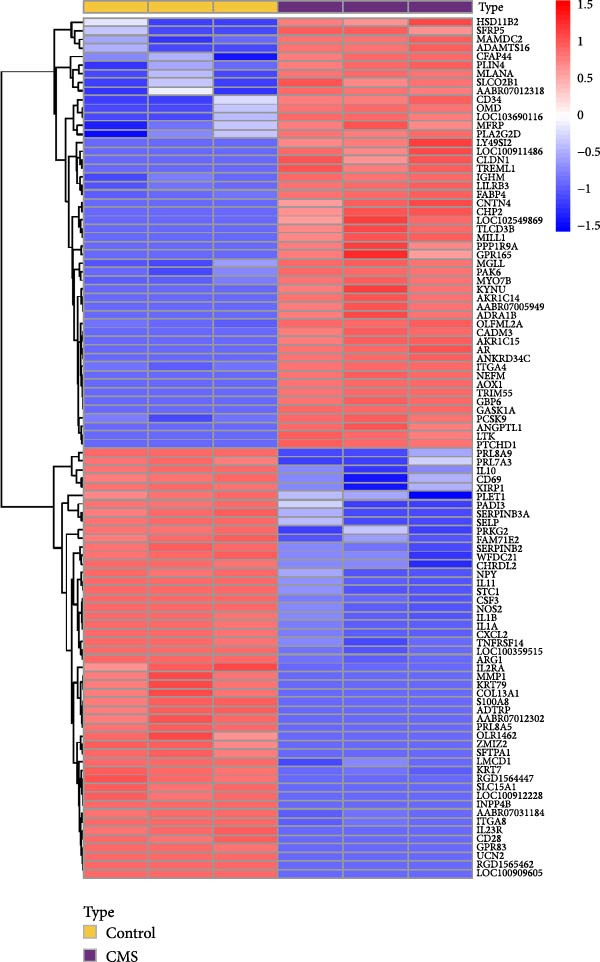
(A)

**Figure:**
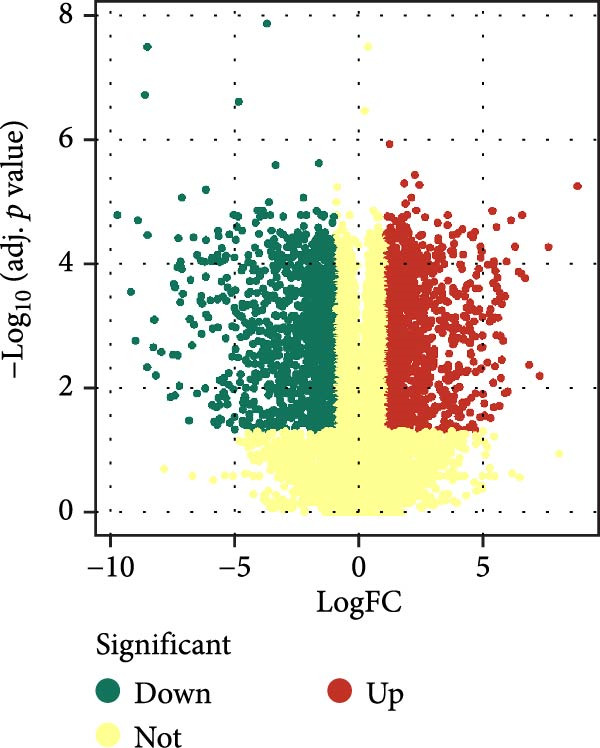
(B)

**Figure:**
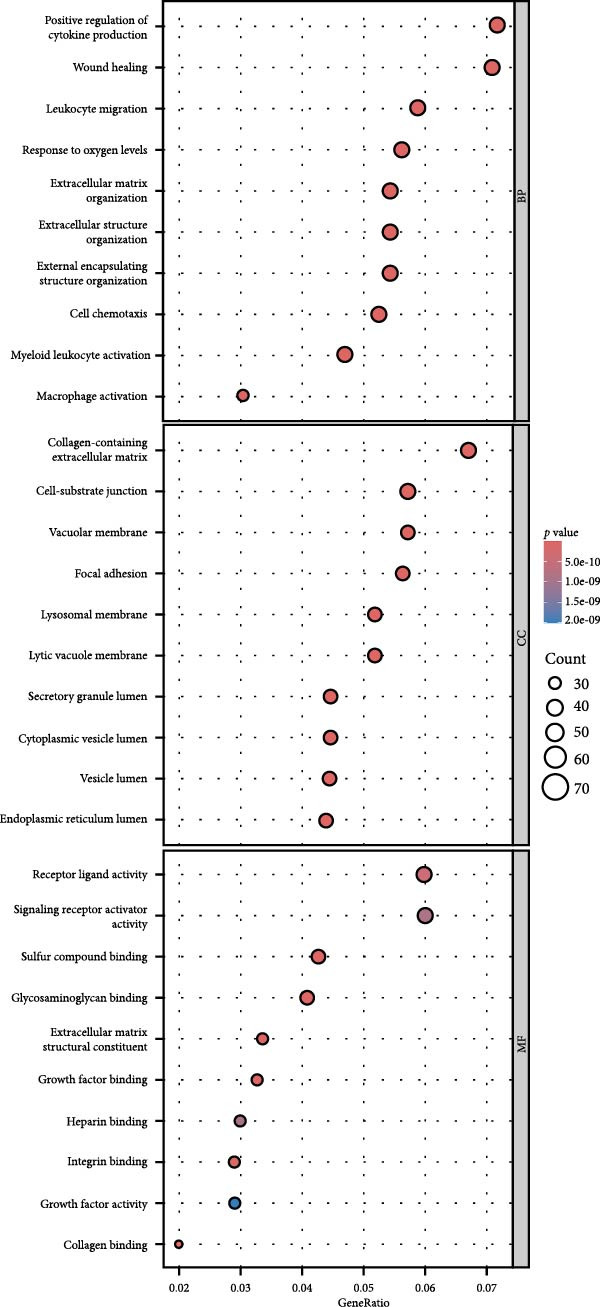
(C)

**Figure:**
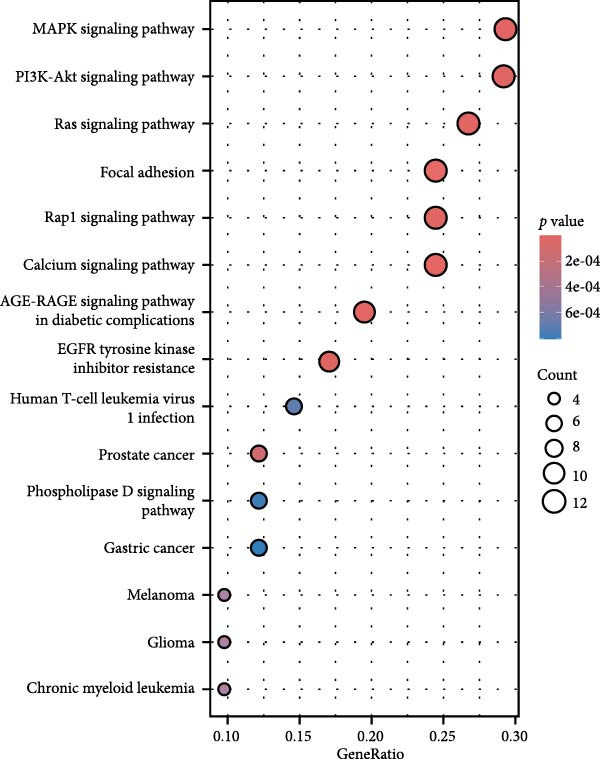
(D)

**Figure:**
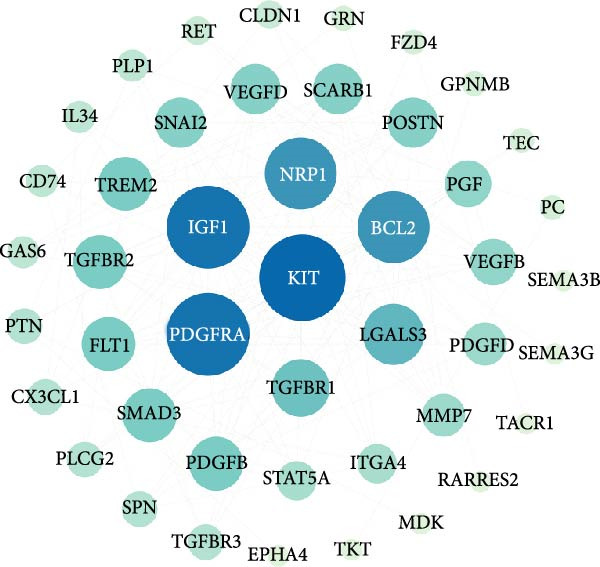
(E)

**Figure:**
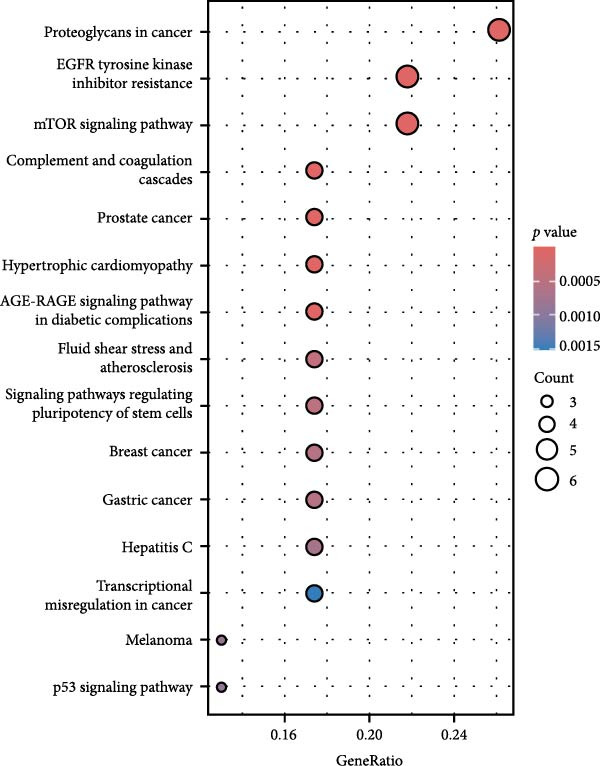
(F)

**Figure:**
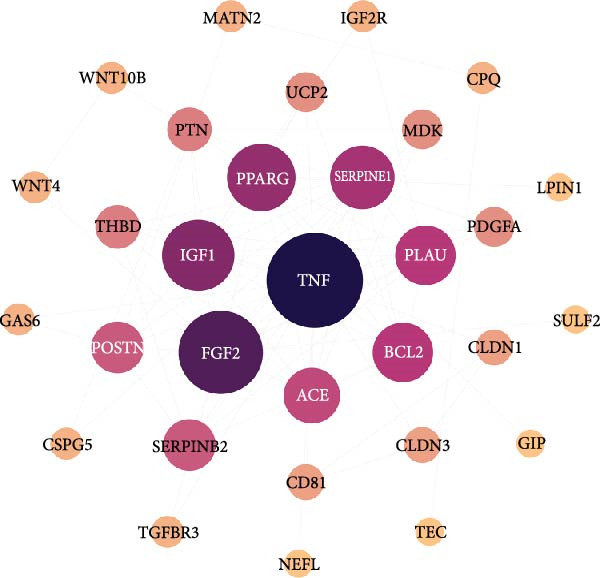
(G)

Genes related to cell migration were analyzed by KEGG pathway analysis and PPI analysis. The differential genes related to cell migration were mainly enriched in the *MAPK signaling pathway*, *PI3K-AKT signaling pathway*, *Ras signaling pathway*, *focal adhesion-associated pathway*, *Rap1 signaling pathway*, *calcium signaling pathway*, etc. (Figure 6D). The PPI network was constructed, and the proteins that were considered to be at the more central part of the PPI network after sorting according to Degree were *KIT*, *PDGFRA*, *IGF-1*, *NRP1*, *BCL2*, *LGALS3*, *TGF-βR1*, etc. (Figure 6E). The genes enriched in wound healing were subjected to KEGG pathway analysis and PPI analysis. Cell migration‐related differential genes were mainly enriched in *proteoglycans in cancer-related pathway*, *EGFR tyrosine kinase-related pathway*, *mTOR signaling pathway*, and *complement and coagulation cascades-related pathway*, etc. (Figure 6F). Proteins that are more central to the protein network include *TNF*, *FGF-2*, *IGF-1*, *PPAR γ*, *SERPINE-1*, *PLAU*, *BCL2*, and *ACE* (Figure 6G).

## 4. Discussion

The EG group that combined autologous fat flap grafting and internal expansion was significantly higher than the other three control groups in terms of dermal cell proliferation capacity as well as collagen secretion capacity. This suggests a synergistic effect between our subcutaneous fat and the mechanical stress generated by internal expansion, which together promoted skin growth in the expanded area. Combined with the previous findings that tissue stiffness recruits stem cells from the somatic circulation to promote adipose regeneration, we suggest that there may be a correlation with ADSCs from the adipose flap [12, 16–18].

ADSCs tracer experiments suggested to us that internal expansion could greatly promote the migration of ADSCs from within the compressed fat flap to the dermis of the skin in the expanded area. A high number of GFP^+^ ADSCs could be observed in the perivascular distribution of the superficial dermis on day 7, and GFP fluorescence signal could be observed in some of the vessels. This result suggests that the migration of ADSCs is likely to be accomplished through the microvascular connection between the fat flap and the dermis. In combination with immunofluorescence staining on day 14 of the EG group, an increase in the number of CD31‐labeled vascular endothelial cells was observed. We further hypothesized that the increase in angiogenesis after tissue compression may promote the formation of a denser vascular network between adipose and dermis, which provides more favorable conditions for the transport and migration of stem cells.

Some of the previous studies have proposed the concept of a “stem cells pool.” In the human body, the subcutaneous fat layer, preconstructed by a single high‐quality fat graft, serves well as an adjacent “adipose stem cells pool.” The ability of stem cells to promote skin regeneration has been proven in some studies [13]. Stimulated by chemical signals or tissue stiffness signals, these mesenchymal stem cells in the pool can be mobilized to participate in tissue regeneration processes such as cell proliferation and collagen secretion [14]. Moreover, compared to therapies that give exogenous stem cells directly, it is safer to mobilize endogenous stem cells for tissue regeneration and repair through tissue engineering strategy.

The role of transient response of epidermal stem cells after being stretched in skin expansion has been demonstrated [19]. Our study confirmed the migration of adipose stem cells and observed a difference in the growth rate of skin with and without subcutaneous fat flaps when subjected to stretch. This suggests that ADSCs may also be subjected to mechanical stretch, thereby participating in the process of skin expansion regulation. By making ADSCs express GFP proteins and observing the destination of ADSCs within the fat flap, we found that there were a large number of GFP^+^ ADCSs distributed in the dermis of the EG group on day 14, in contrast to the FGCG group, in which few GFP^+^ ADCSs were visible in the dermis. This suggests to us that tissue expansion can promote the migration of ADSCs within the compressed fat flap to the dermis of the skin in the expanded area. However, the pathway through which ADSCs migrate directionally to the skin is not precisely determined at present. A high number of GFP^+^ ADSCs were observed to be distributed around blood vessels in the superficial dermis on day 7, and GFP^+^ signals were observed in some of the vessels. This result suggests that the migration of ADSCs is most likely accomplished through the microvascular connection between the fat flap and the dermis. However, more validation may be needed to answer the question of whether this is the main pathway for ADSCs to migrate from the adipose flap.

**Figure 7:**
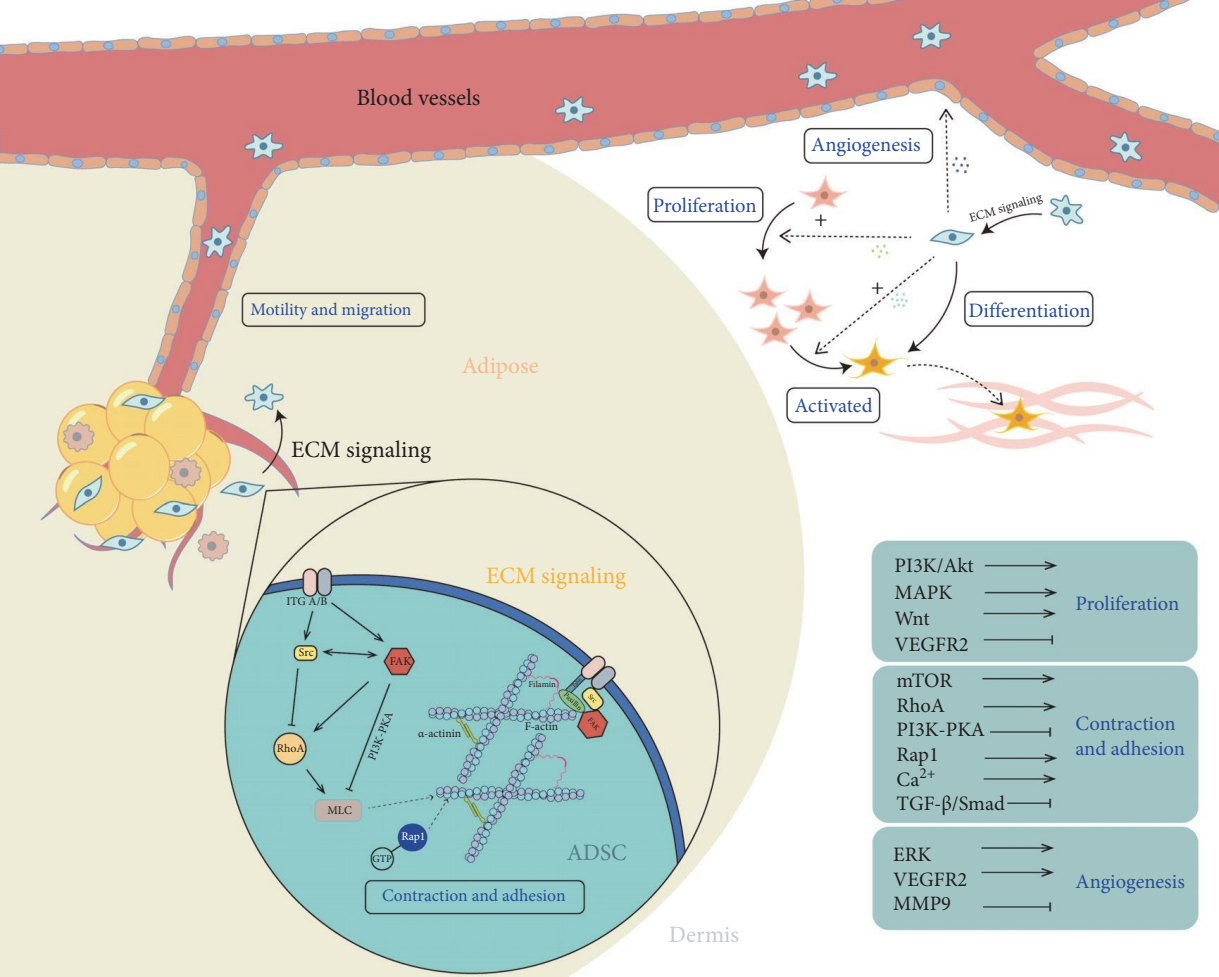
Schematic representation of predicted mechanisms and pathways based on experimental and transcriptomic analysis.

Directional migration of stem cells is regulated by many factors [15, 20–22]. In this model, it is interesting to see what factors regulate the outward migration of some stem cells from the subcutaneous fat flap by “extrusion” and their massive migration toward the dermal tissue in the expanded area. Transcriptomic analysis of MSCs with and without CMS treatment was performed in the hope of predicting possible pathway models for this phenomenon. The analysis yielded DEGs between the two groups, and GO enrichment of these genes revealed that most of these genes are associated with wound healing and migration. Through further searches and analysis in the KEGG database, we found that stem cells subjected to CMS may regulate cytoskeletal‐related proteins such as Integrin, F‐actin, β‐catanin, talin‐1, talin‐2, and vinculin through mechanosignal initiation of the RhoA pathway, PI3K/Akt pathway, and Rap1 pathway, thereby upregulating the cytoskeletal‐associated proteins such as Integrin, F‐actin, β‐catanin, talin‐1, talin‐2, and vinculin, thereby upregulating cell contraction and adhesion (Figure 7).

Alterations in the adhesive capacity of the cells themselves may affect the directional migration properties of the cells. The conventional view is that most normal cells have durotaxis, which means cell migration toward increasing substrate stiffness [23]. However, with many in‐depth studies and theoretical models, such as motor‐clutch‐based modeling, perhaps cell migration is not simply stiffening, but rather toward optimal tissue stiffness as determined by cell‐intrinsic properties (toward cell‐intrinsic “optimal stiffness”) [19, 24–26]. This property is related to the contractile and adhesive functions of the cell. The cell‐intrinsic “optimal stiffness” can be altered by inhibiting or promoting cell contraction and adhesion [27]. This may be related to the regulation of directional cell migration [28]. However, due to the different fundamental properties of different cells, the fate of cells regulated by mechanical force is also different [29]. This implies that there are differences in the regulatory endpoints of different levels of mechanical force signals on the intrinsic contractile adhesion properties of ADSCs cells, and perhaps there exists an optimal stiffness to maximize the mobilization of stem cells within the adipose flap for migration towards the dermis. In addition to this, Chu et al. [30] illustrated that epidermal stem cells can express chemokines in response to mechanical stretch to recruit macrophages and produce growth factors such as IGF‐1 to promote hair growth. Therefore, whether skin tissue is actively involved in the recruitment of ADSCs through certain pathways after being subjected to expansion stretch also needs to be further explored.

In addition, our analysis showed that DEGs in mechanically affected stem cells were highly enriched in terms of “cytokine production, wound healing, collagen secretion” and other terms related to tissue regeneration and wound healing, which is consistent with the phenomenon observed in our study. Through further pathway enrichment and protein interactions analysis, it was suggested that the core cytokine pathways were MAPK pathway, PI3K/Akt pathway, Wnt signaling pathway, and mTOR pathway, which were closely related to cell proliferation and anti‐apoptosis ability. Among them, the mTOR pathway plays an important role in regulating the expression of VEGF and FGF to promote angiogenesis and collagen synthesis. In addition, the activation of PPARγ pathway and Wnt signaling was associated with active metabolic functions.

Overall, our study further supports that fat layer construction prior to skin expansion helps to promote skin growth. This promotion may be due to the migration of ADSCs from adipose to dermis by compression, and ADSCs migrating to dermis further promote skin stretching through direct differentiation or paracrine cytokines to promote cell proliferation and collagen synthesis. This finding suggests that in clinical tissue expansion, prioritizing subcutaneous adipose grafting prior to initiating expansion procedures following confirmation of adipose viability may optimize therapeutic efficacy. However, the exact clinical efficacy of this strategy needs to be supported by stronger clinical evidence.

This study has several limitations. First, the precise migratory pathways of ADSCs remain undefined and warrant further investigation using advanced methodologies such as intravital imaging or single‐cell tracking. Second, the mechanistic roles of signaling pathways in regulating ADSC behavior under mechanical stress require deeper validation through genetic or pharmacological approaches. Third, the current sampling intervals (7–28 days) may lack temporal resolution to capture early dynamic processes; more frequent time points (e.g., 3–5 days) during the initial expansion phase could enhance the granularity of data. Addressing these limitations in future studies will strengthen mechanistic insights and refine translational strategies for clinical applications.

## 5. Conclusion

The presence of subcutaneous fat and the mechanics of tissue expansion act synergistically in skin growth and collagen production. This may be related to the migration of ADSCs from the adipose flap to the dermis under the expansion. Simultaneously, the observation of fluorescent ADSCs within the skin vasculature suggesting that ADSCs may be migrating via a vascular network localized between dermis and adipose.

**Nomenclature**
ADSCs: Adipose‐derived stem cells
BCG: Blank control group
BPs: Biological processes
CCs: Cellular components
CMS: Cyclic mechanical stretch
DEGs: Differentially expressed genes
EG: Experimental group
FGCG: Fat grafting control group
MF: Molecular function
MSCs: Mesenchymal stem cells
PCNA: Proliferating cell nuclear antigen
PMRT: Postmastectomy radiation therapy
TECG: Tissue expansion control group.

## Conflicts of Interest

The authors declare no conflicts of interest.

## Author Contributions

Haojing Tang and Zhixin Xue contributed equally to this study and should be considered as co‐first authors. Yunjun Liao and Ziqing Dong contributed equally to this study and should be considered as co‐corresponding authors.

## Funding

This work was supported by the National Natural Science Foundation of China (81971852), Nanfang Hospital President’s Fund (2024A005), and Clinical Program of Nanfang Hospital, Southern Medical University (2022CR007).

## Data Availability

The datasets generated and analyzed during the current study are available from the corresponding author upon reasonable request.
